# The effect of virtual reality based disaster training on disaster preparedness of nursing students: a randomized controlled study

**DOI:** 10.1186/s12912-026-04373-w

**Published:** 2026-02-04

**Authors:** Bekir Ertuğrul, Ziyafet Uğurlu

**Affiliations:** 1 Vocational School of Health, First and Emergency Aid Programme, Baskent University, Ankara, Türkiye Turkey; 2 Faculty of Health Sciences, Department of Nursing, Baskent University, Ankara, Türkiye Turkey

**Keywords:** Virtual reality, Simulation, Disaster preparedness, Psychological preparedness, Nursing education, Nursing students

## Abstract

**Objectives:**

The aim of this study is to determine the effect of virtual reality-based disaster preparedness training on the level of disaster preparedness in nursing students.

**Design:**

A pretest, post and follow-up test randomized controlled trial.

**Methods:**

Nursing students (*n* = 67) studying in a nursing department in Turkey from February to April 2024 were included in the study. Data were collected using the “Descriptive Information Form”, “Disaster Preparedness Information Questionnaire”, “Disaster Preparedness Skills Form”, “Disaster Preparedness Scale (DPS)” and “Psychological Preparedness for Disaster Threat Scale (PPDTS)”. In the study, virtual reality simulation was applied to the nursing students in the intervention group in addition to the standard disaster training for 4 weeks, with each session lasting 50 min, while the nursing students in the control group received standard training. Chi-square test, mixed design ANOVA and Bonferroni multiple comparison test were used to analyze the data.

**Results:**

It was determined that there was no statistically significant difference between the intervention group and the control group in terms of disaster preparedness knowledge and skill levels, DPS and PPDTS pre-test mean scores (*p* > 0.05). In the intervention group’s nursing students’ post-test and 8th week follow-up test; disaster preparedness knowledge level showed medium effect (η²=0.112), skill level showed large effect (η²=0.282), DPS score at a medium effect level (η²=0.084), and PPDTS score at a large effect level (η²=0.163). These values were found to be statistically significantly higher compared to the control group.

**Conclusion:**

Virtual reality-based disaster preparedness training was found to be an effective method to improve disaster preparedness levels compared to standard training methods. In this context, it is recommended to use virtual reality technology in disaster preparedness education process and disaster nursing curriculum planning.

**Registration:**

The study has been registered with the National Library of Medicine Clinical Trials: NCT06253156, Registration date: 02 February 2024.

## Introduction

Disasters, which occur worldwide and are generally unpredictable, constitute a global public health problem [1]. The Emergency Events Database, one of the leading international databases recording such events, reports that there have been 7,348 disaster events in the last two decades. Disasters have taken around 1.23 million lives, an average of 60,000 per year, and affected more than 4 billion people in total. Disasters not only cause loss of life on societies but also disrupt the social structure and cause serious economic losses [2].

It is stated that individual preparedness is at least as important as institutional, regional and national preparedness in disasters and emergencies [3]. Although individual preparedness for disasters is not at the desired level in the world, it is also known that it is not at an adequate level in our country. In the process of disaster preparedness, it is necessary to create training programs in order to increase the awareness that will ensure individual preparedness and to create behavioral change [4].

In the process of disaster preparedness, psychological preparedness as well as individual and physical preparedness has been emphasized in recent years because it promotes community resilience [5]. Related studies indicate that the more psychologically prepared individuals are, the higher their physical or material preparedness and resilience [6, 7]. The psychological impact of stress caused by unusual situations or events can negatively affect decision-making and performance. In a study conducted by Roudini et al. [7], it was shown that psychological preparedness for a disaster event contributes to more effective management of the stress experienced, individuals feel more in control at the time of disaster and experience fewer mental health problems after the disaster.

One of the health goals in the Sustainable Development Goals is to empower healthcare workers to improve the quality of healthcare services through applied training [8]. Nurses, who constitute the majority of healthcare workers, are responsible for protecting the physical and mental health of disaster victims at all stages of the disaster [9]. Nurses always have important roles in disaster preparedness, intervention and recovery efforts [10]. However, it is accepted that nurses often do not have sufficient knowledge about disaster preparedness, have low skill competence and are not adequately prepared psychologically against disasters [11–13]. Teaching strategies in nursing programs should be reviewed and evaluated in terms of new and current approaches in disaster nursing for disaster preparedness of nurses [14]. Undergraduate nursing curriculum should be organized in a way to prepare nursing students technically and mentally for the disaster process [15]. Disaster preparedness training is indispensable for nursing students to function independently and to develop their skills to cope with emergencies. However, such trainings are not included much in public health education curricula. In a study examining the curricula of all nursing undergraduate programs in Portugal, it was reported that 85% of the universities did not have any content related to disaster preparedness [16]. It is seen that classical methods such as posters, signs, games, documents, short videos, storybooks, puzzles, computer games, music, poems, dances, puppets, magic, street shows, improvisations, pantomimes, clubs, projects, disaster drills are used more frequently in disaster preparedness trainings [17]. Advances in information technology and innovative developments reveal the necessity of taking into account the personality structure and learning styles of generations in education. Contemporary healthcare education increasingly emphasizes innovative and active learning strategies, with virtual reality offering new opportunities to support experiential and applied learning [18].

One of the innovative methods used in disaster preparedness training is virtual reality (VR)-based programs [19]. VR is a technology that creates a simulated, three-dimensional environment in which users can interact using specialized equipment such as headsets and control devices. It has been reported that integrating VR technology into classrooms has a positive effect on student engagement and learning [20]. It has been reported that students using this technology have higher levels of motivation and engagement and perform better in academic tasks [21]. It is seen that positive results have been obtained in many studies conducted with nursing students in many areas where VR technology was used [22, 23]. However, the use of VR technology in disaster education may be limited due to high equipment and software development costs [24]. In a systematic review on disaster medicine, it is stated that VR technology should be given in addition to traditional methods and more research should be conducted to create an educational model in this field [25]. In the existing literature, although there are limited studies on increasing the intervention capacity of nursing students on the basis of VR in disaster education [26] and providing evacuation management in disasters [27], there are no studies on physical and psychological preparedness for disasters. It is thought that the VR-based disaster education prepared despite the inadequacies seen in the disaster preparedness process and curriculum of nursing students will make a significant contribution to the literature. In addition, the study is very important in terms of providing data to academic units that are willing to incorporate the developing and changing technology based on VR into their curriculum in disaster education and disaster preparedness process. In this study aims to evaluate the effectiveness of VR-based education compared to standard education in the disaster preparedness process for nursing students.

### Hypotheses of the study

The following hypotheses were tested in the study:

- H1: Virtual reality-based disaster preparedness training significantly increases nursing students’ disaster preparedness knowledge levels and disaster and emergency kit preparation skills.
- H2: Virtual reality-based disaster preparedness training significantly increases nursing students’ disaster preparedness levels, including physical and psychological preparedness dimensions.

## Methods

### Study design

This study was conducted with a parallel group randomized controlled experimental design with pretest-posttest, follow-up test. This study has been reported in accordance with the 2017 CONSORT guidelines developed for studies evaluating non-pharmacological interventions [28]. ClinicalTrials.gov registration number of the study: NCT06253156.

### Study setting and participants

The population of the study consisted of 100 nursing students studying in the 3rd and 4th grades of the nursing department of a foundation university in the spring semester of the 2023–2024 academic year. The reason why nursing 3rd and 4th grade students were selected in the study is that they have basic knowledge and equipment about the nursing process [13, 14].

The minimum sample size was determined using the G*Power (V3.1.9.7) program [29]. Repeated measures ANOVA approach was used to calculate the sample size. The sample size calculation was based on the primary outcome, the total Disaster Preparedness Score (DPS). In this calculation, the effect size of a previous study was utilized [26]. Accordingly, the minimum sample size to be reached for 2 groups and 3 repeated measures with a power of 0.80 (1-β error probability), α = 0.05 (type 1 error probability), f = 0.312 effect size was calculated as 56 people in total, 28 people in groups. Considering the possible losses, the sample size was increased by 20% and consisted of a total of 68 people, 34 in intervention and 34 in control groups. Following completion of the intervention process, one participant in the experimental group withdrew from the follow-up process, so the study was analyzed according to protocol with 67 participants (intervention 33, control 34).

#### Inclusion criteria

Being a student in the 3rd or 4th year of nursing at the relevant universities where the study will be conducted, not having taken any disaster-related course before, being willing to participate in the study and having completed the informed consent form.

#### Exclusion criteria

Having any problem with vision, hearing and balance.

#### Exclusion criteria

Individuals who participated in the study wanting to leave the study voluntarily, having health problems before, during and after the use of VR glasses (Migraine, vertigo, active nausea, vomiting, headache, etc.), not wanting to participate in VR-based disaster preparedness training, not participating in the pre-, post- or follow-up test.

### Randomization

**Fig. 1:**
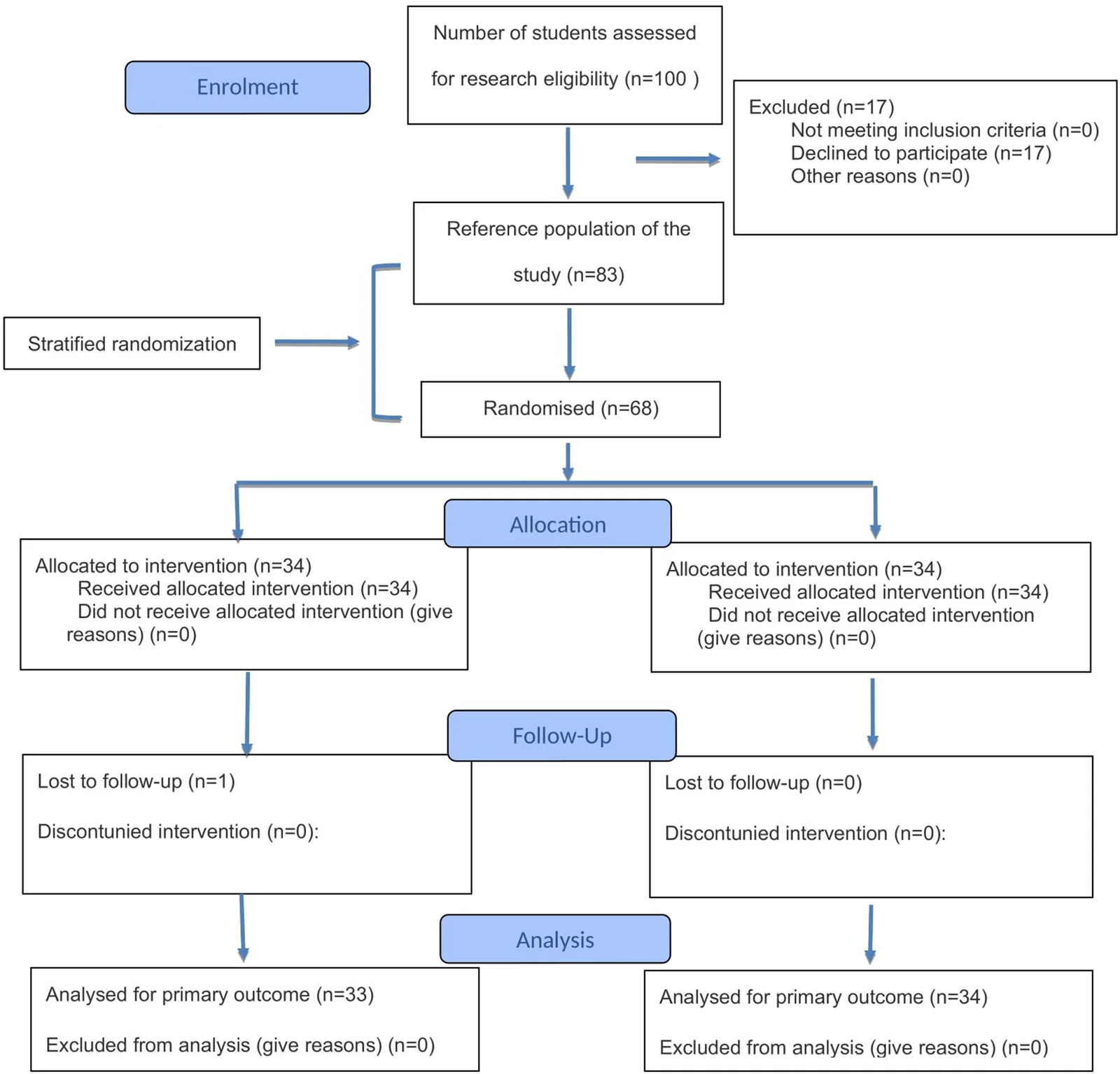
CONSORT flow diagram

During the randomization process of nursing students who agreed to participate in the study, student numbers and the grade they were in were included in the database created instead of their names. The number of students evaluated for eligibility in the study (*n* = 100) was taken into consideration for inclusion and exclusion criteria and the study population (*n* = 83) was determined. Random assignment was created using stratified randomization method in order to prevent differences that may occur in the intervention (students receiving VR-based disaster preparedness training + standard disaster training) and control (students receiving standard disaster training) groups. In stratified randomization, a separate block is generated for each covariate combination and participants are assigned to the appropriate block of covariates [30]. Participants were randomly assigned to groups using stratified block randomisation to maintain a balanced distribution of academic year characteristics. Third and fourth-year nursing students were evaluated as separate strata. Within each stratum, participants were assigned to groups (intervention or control) in a 1:1 ratio. The 34 students in each stratum were distributed as follows: 17 students in the intervention group and 17 students in the control group (a total of 4 groups: 3rd-year intervention, 3rd-year control, 4th-year intervention, 4th-year control).

(Fig. 1). Block randomization was applied throughout the enrollment process to maintain equal group sizes within each stratum. The randomisation sequence was generated prior to the commencement of the study, and group assignments were kept confidential until participant assignment. The randomization process was determined by an independent (blind) statistician according to student numbers and no intervention was made. The rankings of the students randomly assigned to the intervention and control groups according to student numbers were shared only with the researcher by an independent statistician.

### Blinding

Due to the nature of the intervention, it was not possible to blind the participants and the researcher. However, to reduce performance bias, a specialist teaching staff member with teaching experience in nursing and disaster management was blinded during the assessment of the skill cards. Furthermore, during the analysis process, the statistician worked without access to group information, thereby ensuring blinding. These blinding procedures were considered a methodological strength of the study and were implemented to enhance internal validity.

### Instruments

The data of the study were collected by using the Descriptive Information Form, Disaster Preparedness Information Questionnaire, Disaster Preparedness Skills Form, Disaster Preparedness Scale (DPS) and Psychological Preparedness for Disaster Threat Scale (PPDTS).

#### Introductory ınformation form

The Descriptive Information Form was developed by the researcher as a result of the review of the studies on disaster and VR application in the literature [31–33]. This form included 21 questions about the participants’ socio-demographic characteristics such as age, gender, etc. and disaster preparedness such as previous disaster experience, type of disaster experienced, knowledge about emergency/disaster preparedness plan, etc.

#### Disaster preparedness ınformation questionnaire (DPIQ)

Disaster Preparedness Information Questionnaire (DPIQ) was created by the researcher by examining the studies conducted in the field of disaster in order to determine the disaster preparedness of nursing students [34–36]. In the disaster preparedness process, there were 40 multiple-choice questions with 4 options, with 10 questions for each topic for the 4 topics planned to be given in determining the knowledge levels of the participants. Each question from the Disaster Preparedness Knowledge Questionnaire is 1 point, totaling 40 points. The increase in the score indicates an increase in disaster preparedness. For the DPIQ, expert opinion was obtained from 8 faculty members with doctorate degrees in Public Health Nursing, Psychiatric Nursing, Surgical Nursing, Public Health, Psychology and Disaster. Lawshe Technique was used for the content validity of the question form. Content Validity Index (CVI) values were calculated after expert opinions. The CVI value of the expert opinions was found to be 0.93.

#### Disaster preparedness skill form (DPSF)

In order to evaluate the disaster and emergency kit preparation skills of nursing students, which is an important stage in the disaster preparedness process, the disaster and emergency kit preparation checklist of the Disaster and Emergency Management Presidency was taken as a reference and thus standardization was ensured in the evaluation process [37, 38]. The number of correct answers given by nursing students was evaluated by an independent researcher by giving 1 point to each correct material category. The lowest score on the DPSF was 0 and the highest score was 25. The increase in the number of materials written by the nursing students on the skill form shows that their disaster preparedness skill levels increased. The suitability of the DPSF was evaluated by 5 experts with competence in Public Health Nursing, Psychiatry and Disaster. Lawshe Technique was used to evaluate the content validity of the DPSF. The CVI value of the expert opinions was found to be 1.

#### Disaster preparedness scale (DPS)

Disaster Preparedness Scale (DPS) was developed by Şentuna and Çakı (2020) to determine disaster preparedness [39]. The scale consists of 13 items and 4 sub-dimensions: “disaster physical protection”, “disaster planning”, “disaster relief” and “disaster warning systems”. The scale is in 4-point Likert type as “1-Strongly No”, “2-No”, “3-Yes”, “4-Strongly Yes”. The lowest score that can be obtained from the scale is 13 and the highest score is 52. The higher the score obtained from the scale, the higher the level of disaster preparedness. Cronbach’s Alpa coefficient calculated for the whole scale is 0.82. In this study, Cronbach’s alpha value was 0.82 for pretest, 0.86 for posttest, and 0.88 for follow-up test.

#### Psychological preparedness for disaster threat scale (PPDTS)

The scale was adapted into Turkish by Türkdoğan Görgün et al. (2023) [40]. The scale, which aims to measure the level of psychological preparedness against disaster threats, consists of 21 items. It consists of three sub-dimensions: Knowledge and Management of External Situational Environment (9 items), Management of Emotional and Psychological Reactions (9 items), Management of Social Environment (3 items). The scale is a 4-point Likert scale ranging from strongly disagree (1) to strongly agree (4). The lowest score is 21 and the highest score is 84. The Cronbach Alpha reliability coefficient of the scale is 0.95, while the sub-dimensions are 0.91, 0.93 and 0.83 respectively. In this study, the Cronbach Alpha reliability coefficient of the scale was 0.92 for pretest, 0.95 for posttest, and 0.94 for follow-up test.

### Data collection

This study was conducted in three phases: (1) preparation of the lesson, case and simulation scenario; (2) pilot study; and (3) actual study.

#### Preparation stage

Disaster preparedness standard course training content was prepared as a result of existing literature reviews for disaster preparedness process [32, 41, 42]. In the prepared training content; Disasters, Determination of Non-Structural Risks and Taking Measures (Early Warning Systems, Life Triangle, Collapse-Catch-Hold, Hazard Hunt), Preparation of Family Disaster Plan, Importance, Equipment and Preparation of Disaster and Emergency Kit, Psychological Preparation for Disaster Threat were included. Nine expert opinions were taken for the prepared training content. Expert opinions were evaluated with Lawshe technique and the CVI was 0.96. In the process of preparing the VR based disaster training, the existing literature was reviewed for the disaster training topics planned to be given as a design and the visuals to be used on the basis of VR were provided. In the development of VR based disaster training, a computer engineer with experience in the field of software was worked with. The VR application has been developed by an expert in the field using industry-standard tools to ensure realism and functionality. In the educational design, interactive elements that support experiential and active learning, environmental realism, and decision-making processes have been prioritised over technical details [43]. The virtual reality-based disaster training application was designed for physical preparedness in a virtually created home environment and was implemented over four sessions. In the first session, students identified non-structural risks in the home and implemented mitigation measures. In the second session, students were taught how to prepare a disaster and emergency kit and prepared the kit practically in the virtual environment. In the third session, family disaster plan checklists were reviewed and steps related to implementing the plan were evaluated. In the fourth session, breathing exercises, positive thinking techniques and psychological preparedness practices were carried out in a virtually created natural environment. Each session was designed to support student interaction and learning processes, and the implementation process was monitored using observations and checklists. Participant compliance was monitored through session attendance and task completion levels; individuals who did not meet the predefined compliance criteria were assessed as non-compliant. Eight expert opinions were taken for the prepared training content. HEMDA G/I, which is an evaluation tool for audio-visual materials, was used to evaluate the expert opinion. According to the HEMDA G/I results, the level of comprehensibility was 98% and the level of applicability was 100%.

#### Pilot study

A pre-application was conducted to evaluate the suitability, applicability and comprehensibility of the prepared training materials and data collection tools to the target group. Since the information in the form was found to be understandable, no changes were made. The average response time of the data collection tool was 50 min. For the applicability of the developed disaster preparedness simulation, a pre-application was conducted with students and instructors. The average time to be given to the students for each section created in the disaster preparedness simulation was determined. As a result of this evaluation, it was seen that an average of 10 min for the 1st section, 5 min for the 2nd section, 5 min for the 3rd section and 5 min for the 4th section would be sufficient. No changes were made as a result of the feedback received after the simulation pre-application. Students participating in this process were excluded from the scope of the study.

#### Actual study

The intervention implementer was trained according to pre-determined rules and followed a written guide, ensuring that the intervention was applied consistently to all participants. Implementation fidelity was measured to assess whether the programme was implemented as planned and whether the intervention was delivered in accordance with its purpose. Adequate compliance is defined as participation in at least 3 of the intervention sessions (3 out of 4 sessions, 75%). All participants were included in the analysis, and no participants were excluded due to non-compliance. The study was conducted in the following stages.

Step 1. After determining the research groups through randomization, the necessary ethical approval and institutional permissions were obtained. After this process, the purpose of the study was explained to the nursing students assigned to the intervention and control groups and information meetings were held about the road map to be followed by each group.

Step 2. Consent was obtained from the nursing students in the intervention and control groups, and then the pre-test of the Introductory Information Form, DPS, PPDTS and Disaster Preparedness Knowledge Question and Skill Form were administered. In the evaluation of the disaster preparedness skill levels of nursing students, their ability to prepare a disaster and emergency kit was tested. In this process, an independent researcher, selected apart from the researcher, gave blank cards and envelopes to the participant nursing students and asked them to write their student numbers on these cards. The information about which group the students were in was not disclosed to the independent researcher. The independent researcher asked the students to write the materials that should be included in the disaster and emergency kit and to put these cards in envelopes and deliver them. In the evaluation of the disaster preparedness knowledge questions, the researcher gave optical forms and 40 questionnaires to the nursing students. The students were asked to write only their student numbers while filling in the optical forms and were informed that each question had only 1 correct answer and that wrong answers would not affect the correct results.

Step 3. The nursing students in the intervention and control groups received standardized disaster preparedness training for 4 weeks, one session per week, each session lasting 50 min. In this training, the researcher used lecture, question-answer and discussion methods. After the standard training, VR-based disaster simulation was applied in the intervention group for the topics determined each week. The control group received no intervention other than standard training. This design was planned to evaluate the effectiveness of VR as an experiential teaching method added to standard education. During the implementation process of the VR simulation, appointment lists were created on the same day according to the availability of the students participating in the study and students were ensured to keep these appointments.

Step 4. At the end of the 4-week training, a post-test was administered to the nursing students in the intervention and control groups. In the post-test, students were administered DPS, PPDTS and Disaster Preparedness Knowledge Question and Skills Forms.

Step 5. In the 8th week of the beginning of the study, the nursing students in the follow-up test intervention and control groups were asked to fill in the DPS, PPDTS and Disaster Preparedness Knowledge and Skills Forms again in accordance with the rules followed in the previous processes (pretest and posttest). This follow-up period was chosen because it is consistent with the time intervals used in similar disaster preparedness training studies in the literature to monitor the effects of training. A total of 33 students from the intervention group (1 student did not want to participate) and 34 students from the control group participated in the follow-up test. After the collection of the research data, a VR-based disaster preparedness training simulation was made for the nursing students in the control group in order to prevent missed opportunities.

### Statistical analysis

The data obtained in the study were computerized using IBM SPSS 27.0 package program. While evaluating the research data, frequency distribution (number, percentage) for categorical variables and descriptive statistics (mean, standard deviation) for numerical variables were given. The conformity of the data to normal distribution was determined using Skewness-Kurtosis (skewness and kurtosis) values [44]. Chi-square analyses (Pearson chi-square test/Fisher’s exact test), independent sample t test and Mixed ANOVA were used to examine the difference between two groups, time intervals and group*time interaction. Bonferroni test was applied to determine from which group the difference originated. In the evaluation of effect size in the study, Eta square was determined as ≥ 0.01 small effect, ≥ 0.06 medium effect, ≥ 0.14 large effect [45]. Cronbach’s alpha value was used for scale reliability. For statistical significance, *p* < 0.05 was accepted.

### Ethical considerations

This study has been approved by the Research Committee and Ethics Committee of Baskent University Medical and Health Sciences (KA23/423, decision numbered 23/206, dated 27/12/2023). The necessary permission was received from the Nursing Department where the research was conducted (decision numbered 311103, dated 05.02.2024). Permission was received via e-mail for the Disaster Readiness Scale and Psychological Preparedness Scale for Disaster Threat to be used in the research. After the nursing students participating in the research were informed, their verbal and written consents were obtained. The research was conducted in accordance with the Principles of the Declaration of Helsinki.

## Results

The descriptive characteristics of the nursing students (*n* = 67) in the intervention and control groups are shown in Table 1. The mean age of the nursing students in the intervention group was 21.73 ± 0.98, and the general grade point average was 2.78 ± 0.27, while the mean age of the nursing students in the control group was 21.62 ± 1.23, and the general grade point average was 2.75 ± 0.42. It was determined that there was no statistically significant difference between the intervention and control groups and that all demographic characteristics of the groups showed a homogeneous distribution (*p* > 0.05) (Table 1).

**Table 1:** Descriptive characteristics of nursing students in the ıntervention and control groups (*n* = 67)

		Intervention Group (*n* = 33)		Control Group (*n* = 34)		Test Statistics	*p*	
		*n*	%	*n*	%			
Classroom	3	16	48.5	17	50.0	χ2 = 0.015*	0.901	
	4	17	51.5	17	50.0			
Sex	Female	30	90.9	31	91.2	χ2 = 0.001**	1.000	
	Male	3	9.1	3	8.8			
Marital status	Single	33	100.0	34	100.0	-	-	
	Married	0	0.0	0	0.0			
Who (someone) lives with	Family	28	84.8	24	70.6	χ2 = 2.112*	0.549	
	Friends	2	6.1	3	8.8			
	Alone	1	3.0	2	5.9			
	Dormitory	2	6.1	5	14.7			
Number of people living in the household	1–2	4	12.1	6	17.6	χ2 = 0.414*	0.813	
	3–4	18	54.5	17	50.0			
	5 or more	11	33.3	11	32.4			
Social security	Yes	31	93.9	34	100.0	χ2 = 2.124**	0.239	
	No	2	6.1	0	0.0			
Longest lived in	District	3	9.1	5	14.7	χ2 = 0.502**	0.709	
	Province	30	90.9	29	85.3			
Income status	Income exceeds expenditure	8	24.2	12	35.3	χ2 = 5.882*	0.053	
	Income equals expenditure	20	60.6	22	64.7			
	Income is less than expenditure	5	15.2	0	0.0			
Mother education level	Primary education	13	39.4	9	26.5	χ2 = 1.370*	0.504	
	High School	16	48.5	19	55.9			
	University and beyond	4	12.1	6	17.6			
Father education level	Primary education	8	24.2	6	17.6	χ2 = 3.321*	0.190	
	High School	19	57.6	15	44.1			
	University and beyond	6	18.2	13	38.2			
Age	X̄±SS (min-max)	21.73 ± 0.98 (20–24)		21.62 ± 1.23 (20–27)		t = 0.403	0.688	
Grade point average	X̄±SS (min-max)	2.78 ± 0.27 (1.99–3.21)		2.75 ± 0.42 (1.70–3.64)		t = 0.339	0.736	

χ2 = chi-squared. * Pearson chi-square. ** Fisher Exact Test. t: Independent sample t test. X̄: Mean. SD: Standard deviation. *p* < 0.05

The characteristics of the nursing students in the intervention and control groups regarding their disaster preparedness are given in Table 2. It was determined that there was no statistically significant difference between the characteristics of the nursing students in the intervention and control groups regarding their disaster preparedness and that the groups showed a homogeneous distribution (*p* > 0.05) (Table 2).

**Table 2:** Characteristics of nursing students in the ıntervention group and control group regarding disaster preparedness (*n* = 67)

		Intervention Group (*n* = 33)		Control Group (*n* = 34)			Test Statistics	p
		n	%	n		%		
Exposure to disaster	Yes	16	48.5	12	35.3		χ2 = 1.198*	0.274
	No	17	51.5	22	64.7			
Type of disaster exposed^a^	Earthquake	15	45.5	11	32.4		χ2 = 3.242**	0.356
	Flood	1	3.0	0	0.0			
	Other	0	0.0	1	2.9			
Information about the disaster preparedness plan	Yes	17	51.5	19	55.9		χ2 = 0.128*	0.720
	No	16	48.5	15	44.1			
Finding disaster preparedness adequate	Yes	0	0.0	2	5.9		χ2 = 2.546**	0.280
	No	22	66.7	24	70.6			
	Partially	11	33.3	8	23.5			
Availability of a disaster and emergency kit	Yes	2	6.1	5	14.7		χ2 = 1.338**	0.247
	No	31	93.9	29	85.3			
State of knowing the assembly point	Yes	7	21.2	10	29.4		χ2 = 0.595*	0.441
	No	26	78.8	24	70.6			
Availability of family disaster plan	Yes	3	9.1	4	11.8		χ2 = 0.128**	1.000
	No	30	90.9	30	88.2			
Status of taking measures for non-structural risks	Yes	8	24.2	6	17.6		χ2 = 0.441*	0.507
	No	25	75.8	28	82.4			
Psychological preparedness for disasters	Yes	0	0.0	2	5.9		χ2 = 5.050**	0.168
	No	19	57.6	22	64.7			
	Partially	7	21.2	8	23.5			
	Unknown	7	21.2	2	5.9			

χ2 = chi-squared. * Pearson chi-square. ** Fisher Exact Test. t: Independent sample t test. a: only disaster survivors answered, *p* < 0.05

Table 3 shows the intra-group and inter-group comparisons of the average disaster preparedness knowledge scores of nursing students in the intervention and control groups. Nursing students in the intervention group scored higher on the post-test (*p* < 0.001) and follow-up test (*p* = 0.025). The Mixed ANOVA confirmed a significant Group*Time interaction (F = 8.163, *p* < 0.001), indicating the intervention produced a medium sustained effect (η2 = 0.112). In the study, the mean scores of disaster and emergency kit preparation skills were evaluated with Mixed ANOVA analysis in terms of groups (intervention and control), time intervals, group and time interactions. Nursing students in the intervention group scored higher in the post-test (*p* < 0.001) and follow-up test (*p* < 0.001) on disaster and emergency kit preparation skills. The Mixed ANOVA confirmed a significant Group*Time interaction (F = 25.551, *p* < 0.001), indicating the intervention produced a large sustained effect (η2 = 0.282) (Table 3).

**Table 3:** Intragroup and inter-group comparison of the mean disaster preparedness knowledge and skill scores of nursing students in the intervention group and control group (*n* = 67)

		Intervention Group Grubu (*n* = 33)	Control Group Grubu (*n* = 34)	t	p	Group	Time	Group*Time	Mean Difference (95% CI)	Mauchly’s Sphericity
		X̄±SS	X̄±SS							
Disaster Preparedness Knowledge Questions (Min:0, Max:40)	Pretest^1^	17.58 ± 4.77	17.97 ± 5.00	-0.330	0.742	F = 7.111 *p* = 0.010 η2 = 0.099	F = 170.378 p = < 0.001 η2 = 0.724	F = 8.163 *p* < 0.001 η2 = 0.112	2.51 (0.63–4.39)	χ²(2) = 6.48, *p* = 0.039^a^
	Posttest^2^	31.79 ± 3.95	26.68 ± 6.11	4.076	< 0.001					
	Follow-up test^3^	29.52 ± 4.59	26.71 ± 5.40	2.288	0.025					
Ability to prepare a disaster and emergency kit (Min: 0, Max: 25)	Pretest^1^	7.33 ± 2.75	7.50 ± 3.07	-0.233	0.816	F = 22.063 *p* < 0.001 η2 = 0.253	F = 78.279 *p* < 0.001 η2 = 0.546	F = 25.551 *p* < 0.001 η2 = 0.282	2.35 (1.35–3.35)	χ²(2) = 5.55, *p* = 0.062
	Posttest^2^	13.79 ± 2.47	9.12 ± 2.26	8.065	< 0.001					
	Follow-up test^3^	11.85 ± 2.74	9.29 ± 2.19	4.217	< 0.001					

X̄: Mean, SD: Standard Deviation, t: t test, p: significance level; F: Mixed ANOVA, η2: partial eta-square, CI: Confidence Interval, χ²(2)= Mauchly’s Test of Sphericity, a: Greenhouse–Geisser correction was applied due to violation of sphericity

In the study, at post-test and follow-up, the intervention group demonstrated significantly higher mean scores than the control group on overall disaster preparedness (DPS), disaster physical protection, disaster planning, and disaster warning systems (Table 4). The Mixed ANOVA revealed significant Group*Time interaction effects for DPS (F = 5.983, *p* = 0.003, η² = 0.084), disaster physical protection (F = 8.291, *p* < 0.001, η² = 0.113), disaster planning (F = 5.626, *p* = 0.006, η² = 0.080), and disaster warning systems (F = 3.643, *p* = 0.032, η² = 0.102), indicating medium sustained effects of the intervention across dimensions. It was determined that the difference between the mean scores of disaster relief sub-dimension was not statistically significant (F = 0.051, *p* = 0.919) (Table 4).

**Table 4:** Intragroup and intergroup comparison of disaster preparedness scale and subscale mean scores of nursing students in the intervention group and control group (*n* = 67)

		Intervention Group Grubu (*n* = 33)	Control Group Grubu (*n* = 34)	t-testi	p		Group	Time	Group*Time	Mean Difference (95% CI)	Mauchly’s Sphericity
		X̄±SS	X̄±SS								
Disaster Preparedness Scale (Min: 13, Max: 52)	Pretest^1^	28.33 ± 5.72	27.32 ± 5.67	0.726	0.471		F = 14.016 p = < 0.001 η2 = 0.177	F = 89.468 p = < 0.001 η2 = 0.579	F = 5.983 *p* = 0.003 η2 = 0.084	3.83 (1.79–5.88)	χ²(2) = 9.55, *p* = 0.008^a^
	Posttest^2^	39.88 ± 5.97	33.47 ± 4.46	4.989	< 0.001						
	Follow-up test^3^	39.18 ± 6.51	35.09 ± 5.08	2.876	0.005						
Disaster Physical Protection (Min:5, Max: 20)	Pretest^1^	10.27 ± 2.44	10.03 ± 2.54	0.400		0.691	F = 13.289 *p* < 0.001 η2 = 0.170	F = 94.657 *p* < 0.001 η2 = 0.593	F = 8.291 *p* < 0.001 η2 = 0.113	1.51 (0.68–2.34)	χ²(2) = 8.63, *p* = 0.013^a^
	Posttest^2^	14.91 ± 2.21	12.26 ± 1.69	5.503		< 0.001					
	Follow-up test^3^	14.61 ± 2.49	12.94 ± 1.70	3.205		0.002					
Disaster Planning (Min: 3, Max: 12)	Pretest^1^	5.76 ± 1.94	5.82 ± 2.32	-0.126		0.900	F = 5.204 *p* = 0.026 η2 = 0.074	F = 81.901 *p* < 0.001 η2 = 0.558	F = 5.626 *p* = 0.006 η2 = 0.080	0.87 (0.10–1.64)	χ²(2) = 6.42, *p* = 0.040^a^
	Posttest^2^	9.55 ± 1.72	7.85 ± 2.20	3.501		< 0.001					
	Follow-up test^3^	9.24 ± 1.80	8.24 ± 2.00	2.162		0.034					
Disaster Relief (Min: 3, Max: 12)	Pretest^1^	8.55 ± 1.84	7.88 ± 1.89	1.456		0.150	F = 4.868 *p* = 0.031 η2 = 0.070	F = 18.137 *p* < 0.001 η2 = 0.218	F = 0.051 *p* = 0.919	-	-
	Posttest^2^	9.82 ± 1.53	9.06 ± 1.61	1.976		0.052					
	Follow-up test^3^	9.76 ± 1.52	9.15 ± 1.65	1.571		0.121					
Disaster Warning and Signals (Min:2, Max:8)	Pretest^1^	3.76 ± 1.00	3.59 ± 1.02	0.686		0.495	F = 16.549 *p* < 0.001 η2 = 0.203	F = 35.734 *p* < 0.001 η2 = 0.528	F = 3.643 *p* = 0.032 η2 = 0.102	0.76 (0.38–1.13)	χ²(2) = 2.83, *p* = 0.242
	Posttest^2^	5.61 ± 1.46	4.29 ± 1.00	4.285		< 0.001					
	Follow-up test^3^	5.58 ± 1.39	4.76 ± 1.21	2.549		0.013					

X̄: Mean, SD: Standard Deviation, t: t test, p: significance level; F: Mixed ANOVA, η2: partial eta-square. CI: Confidence Interval, χ²(2)= Mauchly’s Test of Sphericity, a: Greenhouse–Geisser correction was applied due to violation of sphericity

In the study, at post-test and follow-up, the intervention group demonstrated significantly higher mean scores than the control group on overall PPDTS, knowledge and management of external situational environment, emotional and psychological response management and social environmental management (Table 5). The Mixed ANOVA revealed significant Group*Time interaction effects for PPDTS (F = 12.685, *p* < 0.001, η² = 0.163) and emotional and psychological response management (F = 12.538, *p* < 0.001, η² = 0.162) showed a large effect level, knowledge and management of external situational environment (F = 8.455, *p* < 0.001, η² = 0.115) at a medium effect level, and social environmental management (F = 4.056, *p* = 0.028, η² = 0.059) at a low effect level.

**Table 5:** Intra-group and inter-group comparison of the mean scores of the psychological preparedness scale for disaster threat and sub-dimension total scores of nursing students in the intervention group and control group (*n* = 67)

		Intervention Group Grubu (*n* = 33)	Control Group Grubu (*n* = 34)	t-testi	p	Group	Time	Group*Time	Mean Difference (95% CI)	Mauchly’s Sphericity
		X̄±SS	X̄±SS							
Psychological Preparedness for Disaster Threat (Min: 21, Max: 84)	Pretest^1^	52.48 ± 11.51	52.68 ± 9.74	-0.074	0.942	F = 14.954 *p* < 0.001 η2 = 0.187	F = 102.380 *p* < 0.001 η2 = 0.666	F = 12.685 *p* < 0.001 η2 = 0.163	6.57 (3.17–9.96)	χ²(2) = 31.30, *p* = 0.000^a^
	Posttest^2^	75.24 ± 7.64	62.56 ± 9.06	6.184	< 0.001					
	Follow-up test^3^	71.64 ± 8.28	64.41 ± 8.63	3.495	0.001					
Knowledge and Management of External Situational Environment (Min: 9, Max: 36)	Pretest^1^	23.06 ± 5.09	22.38 ± 4.35	0.587	0.559	F = 18.758 *p* < 0.001 η2 = 0.224	F = 97.857 *p* < 0.001 η2 = 0.601	F = 8.455 *p* < 0.001 η2 = 0.115	3.20 (1.72–4.68)	χ²(2) = 14.30, *p* = 0.001^a^
	Posttest^2^	33.55 ± 3.06	27.71 ± 4.46	6.227	< 0.001					
	Follow-up test^3^	31.55 ± 4.35	28.44 ± 3.86	3.093	0.003					
Emotional and Psychological Response Management (Min: 9, Max: 36)	Pretest^1^	21.15 ± 5.76	22.18 ± 6.09	-0.708	0.482	F = 5.241 *p* = 0.025 η2 = 0.075	F = 73.679 *p* < 0.001 η2 = 0.531	F = 12.538 *p* < 0.001 η2 = 0.162	2.40 (0.30–4.49)	χ²(2) = 20.04, *p* = 0.000^a^
	Posttest^2^	30.94 ± 4.26	25.76 ± 5.15	4.477	< 0.001					
	Follow-up test^3^	29.73 ± 4.87	26.68 ± 4.98	2.535	0.014					
Social Environmental Management (Min: 3, Max: 12)	Pretest^1^	8.27 ± 2.23	8.12 ± 1.92	0.306	0.761	F = 11.031 *p* < 0.001 η2 = 0.145	F = 26.333 *p* < 0.001 η2 = 0.288	F = 4.056 *p* = 0.028 η2 = 0.059	0.96 (0.38–1.54)	χ²(2) = 19.07, *p* = 0.000^a^
	Posttest^2^	10.76 ± 1.46	9.09 ± 1.71	4.290	< 0.001					
	Follow-up test^3^	10.36 ± 1.43	9.29 ± 1.53	2.954	0.004					

X̄: Mean, SD: Standard Deviation, t: t test, p: significance level; F: Mixed ANOVA, η2: partial eta-square. CI: Confidence Interval, χ²(2)= Mauchly’s Test of Sphericity, a: Greenhouse–Geisser correction was applied due to violation of sphericity

## Discussion

### Effects of VR-based disaster preparedness training on knowledge levels and disaster and emergency kit preparation skills

In order for nurses to acquire the necessary knowledge and skills, they must be prepared for potential future disasters during their nursing education [46]. However, previous studies have shown that nursing students’ level of knowledge regarding disaster preparedness is generally inadequate [47, 48]. The findings of this study revealed that VR-based disaster preparedness training significantly increased nursing students’ disaster preparedness knowledge levels and that this effect persisted in both the final and follow-up tests. These results are consistent with previous studies reporting that simulation-based training approaches support knowledge acquisition in disaster education. Emaliyawati and colleagues [49] reported in their quasi-experimental study with undergraduate nursing students that simulation-based education significantly increased their disaster preparedness knowledge levels. Similarly, Hawsawi and colleagues’ [50] study also showed that students who received simulation-based training had higher levels of knowledge and competence regarding disasters compared to the control group.

Many studies conducted on nursing students show that students do not have the disaster and emergency kit preparation needed during the disaster preparation process [51, 52]. In this study, it was determined that the disaster and emergency kit preparation skills of the students who received VR-based disaster preparedness training in the intervention group were significantly higher than those in the control group. In the study conducted by Kafes et al. [53] on disaster victims using a VR-based intervention program, it was stated that, similar to the findings of the study, the ability of individuals in the intervention group to prepare a disaster and emergency kit increased. In another study conducted by Şahan and Dinç [54] to evaluate the effect of simulation-based teaching methods on disaster preparedness in 7th grade middle school students, middle school students who received simulation-based teaching techniques had higher disaster and emergency kit preparation skills in their post-test scores compared to those who received standard training.

### Effects of VR-based disaster preparedness training on disaster preparedness levels

The VR-based training provided to the intervention group significantly increased their level of preparedness for disasters compared to standard training, and this effect persisted in both the final test and the follow-up test. In a study in the literature, Shujuan and colleagues [26] reported that in a randomised controlled experimental study using VR technology to improve nursing students’ disaster preparedness levels, the disaster preparedness levels of students in the intervention group increased significantly when compared to those in the control group. Chang and colleagues [55] noted that emergency service nurses who received chemical disaster training with VR had significantly increased disaster preparedness levels. This finding is consistent with previous studies and suggests that VR is an important technology for enhancing learning in disaster preparedness training.

The VR-based disaster preparedness training provided to nursing students in the intervention group significantly improves disaster physical protection, disaster planning and disaster warning systems compared to the control group. This indicates that VR-based disaster preparedness training comprehensively strengthens the different components of disaster preparedness. These findings are consistent with previous studies in the literature that have demonstrated the effects of simulation-based disaster physical protection, disaster planning, and disaster warning systems. Erkin and colleagues [56] and Aslanoğlu and colleagues [57] reported significant increases in students’ physical protection levels after disaster training. Similarly, Davis and colleagues [58] reported in a simulation-based study that students’ post-test scores on disaster planning increased significantly. Kaplan and colleagues [59] reported that nursing students’ disaster planning levels increased significantly after theoretical training and simulation practice. Furthermore, Molan and Weber’s [60] study emphasised that VR technology facilitates the understanding of early warning and alarm systems for disasters. The lack of significance in the Group*Time interaction in the Disaster Relief sub-dimension indicates that the intervention did not create a differentiating effect on participants’ perceptions of disaster relief over time. This finding suggests that attitudes towards disaster relief may be more stable or less sensitive to short-term interventions.

### Effects of VR-based disaster preparedness training on psychological preparedness

The use of innovative learning and training strategies, such as high-quality simulations, is noted to be important in the psychological preparedness process for disasters [11]. In this study, it was found that the psychological preparedness levels of students who received VR-based disaster preparedness training against disaster threats were significantly higher than those of students in the control group in the final test and follow-up test. This finding demonstrates the importance of VR technology in increasing psychological preparedness for disaster threats. In a study conducted by Rajabi and colleagues [61] in Iran, where university students were prepared for earthquake disasters using VR technology, it was determined that students exposed to VR technology had greater psychological preparedness than those trained using a face-to-face education approach. Similarly, in a study on forest fires conducted by Molan and colleagues [62], people were given an intervention experience that challenged their defence capabilities using VR technology. After the experience, it was determined that participants who used VR technology realised that they needed to be more psychologically prepared for disaster threats.

The study observed that students who received VR-based disaster preparedness training significantly improved their external situational awareness and management, emotional and psychological response management, and social environment management compared to students in the control group. These findings demonstrate that VR technology comprehensively strengthens various components of psychological preparedness for disaster threats. No studies examining the effects of VR technology on social environment management among nursing students were found in the literature, highlighting the uniqueness of this finding within the context of nursing education; therefore, studies conducted in non-nursing groups that evaluated the effects of VR technology in this area were considered for conceptual comparison in this discussion [63–65]. In a study conducted by Ruan [63] on undergraduate students, it was determined that students exposed to VR technology had higher levels of environmental knowledge than the group receiving standard education. Other studies evaluating the effectiveness of VR technology in environmental education for secondary school students indicated that students using this technology understood difficult environmental topics better and established stronger connections with the content [64, 65]. Yıldız and Yıldırım [66] reported that VR-based education increased emotional and psychological response management. Furthermore, Kim and Lee [67] and Ünver and colleagues [68] stated that simulation and application-based education increased social environmental management levels.

These findings reveal the unique pedagogical potential of VR technology in education. The experiential learning approach in disaster preparedness training provides environments and settings that can simulate realistic scenarios, enabling the application of theoretically learned knowledge. This allows for the acquisition of more lasting knowledge and skills. The immersive and psychologically safe environment offered by VR supports students in taking risks without fear of making mistakes and deepening their learning processes. These features enable students to show positive developments not only in their knowledge levels but also in critical areas such as physical skills and psychological management. Furthermore, it is believed that VR-based disaster preparedness training can support the development of preparedness and disaster mindset for emergencies such as disasters by increasing situational awareness, and this aspect is considered to have significant potential for future research. In addition to this situation. In addition to this situation, when evaluating the results obtained from the use of VR technology within the scope of the study, the innovative effect of the applied education model on students should be taken into consideration. It is thought that the novelty may have had a positive impact on many parameters, particularly in individuals exposed to VR technology for the first time, in terms of interest, motivation, and participation in learning. Furthermore, the persistence of positive effects in the intervention group during follow-up measurements indicates that VR-based training not only creates a short-term motivational effect but can also have an impact on more lasting learning and preparation levels. It is suggested that future studies should include longer follow-up periods and repeated measurements to more clearly distinguish the novelty effect.

### Limitations of the study

This study has certain cultural and contextual limitations. The research was conducted in a single country; therefore, the findings may not fully reflect nursing education practices or situations that may be influenced by cultural characteristics in other countries. Furthermore, the implementation of VR-based educational applications requires specific technical infrastructure, hardware, and financial resources. The sustainability of such applications may be challenging in low-resource or technologically limited environments. Therefore, the study’s results may not be directly generalisable to all educational settings. Nevertheless, this research presents important findings regarding the use of VR technology in nursing education and can be considered a model that can be adapted to different cultural and institutional contexts. Discussing the transferability of the findings to global nursing education could strengthen the study’s contribution at an international level. Furthermore, the study only includes data from third and fourth-year nursing students. In this context, the data obtained are based on the students’ own statements, and the fact that the data collection tools used are based on self-reports raises the possibility of response bias. In addition, it was not observed during the research process whether the nursing students obtained information about the disaster preparedness process from other sources or whether they individually conducted additional studies on the subject. The virtual reality application may have a novelty effect. In future studies, implementing methods to control students’ access to non-study information sources and using objective measurement tools alongside self-reports could mitigate the impact of this limitation on internal validity. In this study, the content validity of the DPIQ and DPSF scales was confirmed through expert opinion. However, test-retest reliability and exploratory factor analysis were not applied, which is a limitation of the study. Therefore, it is considered that the results obtained should be interpreted with caution. Future research should prioritise the full psychometric validation of these instruments.

## Strengths of the research

The research will contribute positively to the literature by using VR technology in increasing the disaster preparedness levels of nursing students in line with the expectations of the developing and changing modern age and by conducting it in a randomized controlled design. In addition, this research will make significant contributions to the evaluation of the effectiveness of VR technology in disaster education when integrated with standard education. The findings obtained can be used as a reference in practices and future research aimed at increasing the effectiveness of disaster education by nursing educators and researchers.

In order to prevent possible biases in the study, the randomization process was carried out by taking into account student number and class information. In literature reviews, it is seen that separate studies have been conducted with the scales used in the study. In this study, considering the holistic disaster preparedness approach, physical and psychological preparedness in disaster preparedness was addressed and the two scales were used together to evaluate. Since the amount of materials considered to be placed in the disaster and emergency kit during the preparation process of the DPSF may vary due to situations such as the inability of AFAD to determine a clear list of materials, differences in individual priorities, changes that may occur in the current characteristics of countries, etc., the fact that standardization can be achieved is one of the strengths of the study. In addition, a specialist statistician was blinded during the data coding and reporting phases, and an independent researcher was blinded during the evaluation of the disaster preparedness skills form.

## Conclusion

The results of the current study show that VR-based physical and psychological disaster preparedness training has positive effects on increasing disaster preparedness levels in nursing education. It has been observed that courses conducted with VR have a positive effect on learning and have high retention. It is important to integrate VR-based disaster preparedness training into disaster nursing curriculum programs for nursing students. It can be said that such training methods can contribute to the training of more equipped nurses by supporting not only the transfer of knowledge but also emotional resilience and psychological preparation. In this respect, it is recommended that infrastructure, technical support, software support and classroom environments be provided so that these learning models can be easily used in student education in universities.

## Data Availability

The datasets used or analyzed during the current study are available from the corresponding author on reasonable request, in accordance with institutional data sharing policies.

## Abbreviations

VR: Virtual Reality
DPS: Disaster Preparedness Scale
DPIQ: Disaster Preparedness Information Questionnaire
DPSF: Disaster Preparedness Skill Form
PPDTS: Psychological Preparedness for Disaster Threat Scale
SPSS: Statistical Package for the Social Sciences
