# Correction: The effect of virtual reality based disaster training on disaster preparedness of nursing students: a randomized controlled study

**DOI:** 10.1186/s12912-026-05340-1

**Published:** 2026-09-07

**Authors:** Bekir Ertuğrul, Ziyafet Uğurlu

**Affiliations:** 1 Vocational School of Health, First and Emergency Aid Programme, Baskent University, Ankara, Türkiye, Turkey; 2 Faculty of Health Sciences, Department of Nursing, Baskent University, Ankara, Türkiye, Turkey

**Correction: BMC Nursing (2026) 25:207**

**https://doi.org/10.1186/s12912-026-04373-w**

In this article, the research period stated in the abstract should be February to April 2024, instead of February to April 2025.

The original article has been corrected.

